# Alcohol intake and endogenous sex hormones in women: meta-analysis of cohort studies and Mendelian randomization

**DOI:** 10.21203/rs.3.rs-3249588/v1

**Published:** 2023-08-16

**Authors:** Sandar Tin Tin, Karl Smith-Byrne, Pietro Ferrari, Sabina Rinaldi, Marjorie L McCullough, Lauren R Teras, Jonas Manjer, Graham Giles, Loïc Le Marchand, Christopher A Haiman, Lynne R Wilkens, Yu Chen, Sue Hankinson, Shelley Tworoger, A Heather Eliassen, Walter C Willett, Regina G Ziegler, Barbara J Fuhrman, Sabina Sieri, Claudia Agnoli, Jane Cauley, Usha Menon, Evangelia Ounia Fourkala, Thomas E Rohan, Rudolf Kaaks, Gillian K Reeves, Timothy J Key

**Affiliations:** University of Oxford; University of Oxford; International Agency for Research on Cancer (IARC/WHO); International Agency for Research on Cancer (IARC/WHO); American Cancer Society; American Cancer Society; Skåne University Hospital Malmö, Lund University; Cancer Council Victoria; University of Hawai’i Cancer Center, University of Hawai’i; University of Southern California; University of Hawai’i Cancer Center, University of Hawai’i; New York University Grossman School of Medicine; University of Massachusetts; Moffitt Cancer Center; Harvard T.H. Chan School of Public Health; Harvard T.H. Chan School of Public Health; National Cancer Institute; University of Pittsburgh; Fondazione IRCCS Istituto Nazionale dei Tumori; Fondazione IRCCS Istituto Nazionale dei Tumori; University of Pittsburgh; University College London; University College London; Albert Einstein College of Medicine; German Cancer Research Center, DKFZ; University of Oxford; University of Oxford

**Keywords:** alcohol drinking, sex hormones, oestrogens, androgens, breast cancer

## Abstract

**Background:**

The mechanisms underlying alcohol-induced breast carcinogenesis are not fully understood but may involve hormonal changes.

**Methods:**

We investigated cross-sectional associations between self-reported alcohol intake and serum or plasma concentrations of oestradiol, oestrone, progesterone (in pre-menopausal women only), testosterone, androstenedione, DHEAS (dehydroepiandrosterone sulphate) and SHBG (sex hormone binding globulin) in 45 431 pre-menopausal and 173 476 post-menopausal women. We performed multivariable linear regression separately for UK Biobank, EPIC (European Prospective Investigation into Cancer and Nutrition) and EHBCCG (Endogenous Hormones and Breast Cancer Collaborative Group), and meta-analysed the results. For testosterone and SHBG, we also conducted two-sample Mendelian Randomization (MR) and colocalisation using the *ADH1B* (Alcohol Dehydrogenase 1B) variant (rs1229984).

**Results:**

Alcohol intake was positively, though weakly, associated with all hormones (except progesterone in pre-menopausal women), with increments in concentrations per 10 g/day increment in alcohol intake ranging from 1.7% for luteal oestradiol to 6.6% for post-menopausal DHEAS. There was an inverse association of alcohol with SHBG in post-menopausal women but a small positive association in pre-menopausal women. MR identified positive associations of alcohol intake with total testosterone (difference per 10 g/day increment: 4.1%; 95% CI: 0.6%, 7.6%) and free testosterone (7.8%; 4.1%, 11.5%), and an inverse association with SHBG (−8.1%; −11.3%, −4.9%). Colocalisation suggested a shared causal locus at *ADH1B* between alcohol intake and higher free testosterone and lower SHBG (PP4: 0.81 and 0.97 respectively).

**Conclusions:**

Alcohol intake was associated with small increases in sex hormone concentrations, including bioavailable fractions, which may contribute to its effect on breast cancer risk.

## Background

Alcoholic beverages are commonly consumed in many populations and are known to be causally associated with increased risk of several diseases including breast cancer [1, 2]. The mechanisms underlying alcohol-induced carcinogenesis are not fully understood; the mutagenic alcohol metabolite acetaldehyde may be the causal factor for some cancers such as those of the upper gastro-intestinal tract, but the effect on breast cancer may involve hormonal changes.

Earlier intervention studies have reported an acute increase in serum/plasma concentrations of oestrogens and/or androgens within hours after intake of alcohol [3–8] in pre- and/or post-menopausal women, although others found no significant effect [9–11]. Other intervention studies have also found an increase in sex hormone concentrations after daily intake of alcohol for two to three months [12–15]. Similarly, more recent cross-sectional observational studies have associated habitual alcohol intake with high sex hormone concentrations as well as differences in sex hormone binding globulin (SHBG), a glycoprotein that binds to oestrogens and androgens [16–18].

In the study reported here, we combined data from 14 cohort studies and conducted cross-sectional analyses to provide the most comprehensive evidence to date on the associations of usual alcohol intake with serum or plasma concentrations of oestradiol, oestrone, testosterone, androstenedione, dehydroepiandrosterone sulphate (DHEAS) and SHBG in pre- and post-menopausal women, and with progesterone in premenopausal women only. To examine the potential causal associations with testosterone and SHBG, we also conducted two-sample Mendelian Randomization (MR) and colocalisation analyses.

## Methods

### Observational analyses

Data from the UK Biobank, EPIC (European Prospective Investigation into Cancer and Nutrition) and 12 other studies included in the EHBCCG (Endogenous Hormones and Breast Cancer Collaborative Group) consortium were used.

#### UK Biobank

This is a prospective cohort study involving about 500 000 adults, including over 270 000 women, aged 40–69 years when recruited between 2006 and 2010. At the initial assessment visit, usual alcohol intake was assessed using a touchscreen questionnaire, and blood samples were collected from which serum was prepared and concentrations of hormones and SHBG were measured using chemiluminescent immunoassays. The current analysis included pre-menopausal women, who reported they had not had their menopause (i.e., periods had not stopped), and were younger than 50 years of age, and post-menopausal women, who reported they had gone through menopause, or were 55 years or older, or reported a bilateral oophorectomy; those who had a prior history of cancer ((except for non-melanoma skin cancer) or reported currently using hormone therapy (hormone replacement therapy (HRT) and/or oral contraceptives (OCs)) were excluded. Detailed information on the study design and methodology [19], calculation of alcohol intake in grams per day [18] and the assay data [20] has been reported elsewhere.

#### EPIC

This is a prospective cohort study involving about 520 000 adults, including over 360 000 women, aged 25–70 years when recruited from 23 centres across 10 European countries between 1992 and 2000. Diet, including usual alcohol intake, was measured by country-specific questionnaires that were validated against reference measurements based on twelve 24-hour diet recall interviews [21]. Blood samples were collected from about 74% of the participants. The current analysis included pre- and post-menopausal women from nested case-control studies on breast, ovarian, endometrial, cervical, liver and thyroid cancer risk for whom serum (in most of these studies) or plasma concentrations of sex hormones and SHBG were measured. Both pre-cases (women who were cancer-free at the time of blood collection but were subsequently diagnosed with the cancer of interest during follow-up) and controls were included, except for the liver cancer study where only controls were included. Participants were categorised as pre-menopausal if they reported regular menstrual cycles over the 12 months prior to blood collection or were younger than 42 years at recruitment, and as post-menopausal if they reported having had no menses over the past 12 months, were older than 55 years, or reported a bilateral oophorectomy. Women who reported currently using hormone therapy (HRT and/or OCs) were excluded, as well as those from Greece (due to a restriction concerning information governance). Detailed information on the study design and methodology [22], calculation of alcohol intake in grams per day and the assay data [23] has been reported elsewhere.

The EPIC study data for breast cancer were included in the EHBCCG but the EPIC data were analysed separately here because, since the publication of the collaborative analyses, more nested case-control studies of other cancer sites have been conducted and hormone assay data are now available for a larger sample of women.

#### EHBCCG:

Of the seven prospective studies of pre-menopausal women included in the collaborative analysis,[16] three with available information on usual alcohol intake were included: Nurses’ Health Study II (NHS-II), USA; New York University Women’s Health Study (NYU WHS), USA; and the Study of Hormones and Diet in the Etiology of Breast Tumors (ORDET), Italy. Of the 18 studies of post-menopausal women, 11 were included: Cancer Prevention Study-II Nutrition Cohort (CPS-II Nutrition Cohort), USA; Malmö/Umeå, Sweden; the Melbourne Collaborative Cohort Study (MCCS), Australia; the Multiethnic Cohort (MEC), USA; Nurses’ Health Study (NHS I), USA; NYU WHS, USA; ORDET, Italy; Prostate, Lung, Colorectal, and Ovarian Cancer Screening Trial cohort (PLCO), USA; Study of Osteoporotic Fractures (SOF), USA; United Kingdom Collaborative Trial of Ovarian Cancer Screening (UKCTOCS), UK; and the Women’s Health Initiative, Observational Study (WHI-OS), USA. Women who reported currently using hormone therapy (HRT and/or OCs) were excluded.

Details of the individual studies included in this analysis are presented in the Supplementary Materials. These include: references for component studies within EPIC and EHBCCG (Supplementary Table S1), number of women who contributed to each hormone analysis (Supplementary Table S2), measurement of usual alcohol intake (Supplementary Table S3), and blood sample (serum vs. plasma), type of assay and coeffcients of variation for the measured hormones and SHBG (Supplementary Table S4). In all studies, concentrations of free oestradiol and testosterone were calculated from those of total oestradiol and testosterone respectively and of SHBG, assuming that the binding of these hormones to serum SHBG and albumin follows the law of mass action [24]. As albumin concentration was not measured in EPIC and EHBCCG, it was assumed to be constant at 40 g/L [25].

#### Statistical analysis

Analyses were undertaken separately for pre- and post-menopausal women in UK Biobank, EPIC and EHBCCG. STATA 17 (StataCorp, College Station, Texas) was used for all analyses.

Hormone concentrations were logarithmically transformed. In pre-menopausal women, concentrations were standardised for phase of the menstrual cycle (early follicular, late follicular, mid-cycle, early luteal, mid-luteal and late luteal) with residuals from the mean for each cycle phase. The cycle phase was determined using forward dating (UK Biobank [18]), or both forward and backward dating with the latter used where possible (EPIC [26] and EHBCCG [16]).

For each study, hormone concentrations and 95% confidence intervals (CIs) per 10 g/day (approximately one standard drink/day) increment in alcohol intake were estimated using multivariable linear regression models, adjusting for individual component studies (EPIC and EHBCCG), case-control status (EPIC and EHBCCG), age at blood collection (in 2-year categories for pre-menopausal women and 5-year categories for post-menopausal women), previous alcohol use among non-current drinkers (UK Biobank and EPIC), smoking (never, former, current), body mass index (BMI) (< 22.5 kg/m^2^, 22.5–24.9 kg/m^2^, 25–27.4 kg/m^2^, 27.5–29.9 kg/m^2^, 30–34.9 kg/m^2^, ≥ 35 kg/m^2^), number of full-term pregnancies (0, 1, 2, 3, 4+), past use of hormone therapy (HRT and/or OCs; yes/no), age at menopause (in 3-year categories; post-menopausal women only) and menopausal type (natural, surgical; post-menopausal women only). The study-specific results were then pooled using fixed-effect meta-analysis. Potential differences in the estimates by menopausal status were assessed using the Chi-square test for heterogeneity.

In pre-menopausal women, subgroup analyses were undertaken for total oestradiol, oestrone, progesterone and total testosterone by phase of the menstrual cycle (follicular, mid-cycle and luteal). In both pre- and post-menopausal women, subgroup analyses were undertaken for total oestradiol, oestrone and total testosterone by type of the assay used (direct, extraction and mass spectrometry); the individual studies that contributed to each assay type are presented in Supplementary Table S5. Sensitivity analyses were undertaken by restricting the sample to those who reported alcohol intake of < 15 g/day, to those who reported intake of < 30 g/day (i.e. excluding heavy drinkers), and also to those whose blood samples were collected during an ovulatory cycle (progesterone concentrations measured in the mid-luteal phase ≥ 12.72 nmol/L (~ 400 ng/dL) [27].

### MR and colocalisation analyses

#### Data on alcohol intake

A genetic instrument in the *ADH1B* (Alcohol Dehydrogenase 1B) gene (rs1229984) for self-reported alcohol intake (number of drinks per week) was extracted from a GWAS (genome-wide association study) meta-analysis undertaken by the GWAS and Sequencing Consortium of Alcohol and Nicotine Use (GSCAN) [28]. This variant was used due to its highly biologically plausible association with alcohol intake [29]. The minor A allele of this variant increases the activity of *ADH1B* that oxidises ethanol to acetaldehyde, resulting in unpleasant reactions and limiting further drinking [30]. While this polymorphism is less common in people of white European ancestry with a frequency of < 5% (cf. 90% in East Asians), it is nonetheless a strong genetic predictor of alcohol intake in this population [30]. Estimates were available per one SD (approximately 9 drinks/week) increment in alcohol intake and extracted from the GWAS meta-analysis excluding the UK Biobank (*n* = 226 223) to avoid sample overlap between the GWAS for alcohol intake and that for hormone concentrations. The *ADH1B* variant explains 0.19% of the variance in alcohol intake.

#### Data on testosterone and SHBG:

Summary statistics for the association of rs1229984 with SD increments in the concentrations of hormones and SHBG were obtained from a publicly available GWAS of all women, irrespective of menopausal status, from the UK Biobank, extracted from the OpenGWAS platform [31] (dataset used for total testosterone: ieu-b-4864 involving 199 569 women; free testosterone: ieu-b-4869 involving 180 386 women; and SHBG: ieu-b-4870 involving 214 989 women). Data on oestradiol were available but were not used due to the potential limitations related to measurement of this hormone in the UK Biobank (see details in the Discussion); data on the other sex hormones were not available.

#### MR analyses

MR assesses the associations between exposure(s) and outcome(s) using genetic variants associated with the exposure of interest as instrumental variables. A Wald ratio was calculated using the “TwoSampleMR” [32] package in R. To be able to present the MR results in a way which is directly comparable to the observational results, assuming that one standard drink contains 10 g of alcohol, the β estimates generated from the Wald ratio (per one SD increment in alcohol intake) were converted to the estimates per 10 g/day increment. The results were then multiplied by 0.341 (assuming that, for a normal distribution, one SD is 34.1% of the range) to convert the difference in hormone concentrations from units expressed as SD to percentages.

##### Colocalisation analyses:

Colocalisation assesses the probability that two traits are affected by the same genetic variants at a given locus. Using the *ADH1B* variant, colocalisation analyses were conducted to identify the presence of a shared causal locus between alcohol intake and concentrations of testosterone and SHBG where a conventionally significant association was observed in MR analyses. The “coloc” package [33] in R was used to estimate the posterior probability for two traits sharing the same causal variant (PP4) in a 150 kb LD (linkage disequilibrium) window centred on rs1229984, with PP4 > 0.70 corresponding to strong evidence of colocalisation [34]. Priors chosen were: p1 = 10^−3^, p2 = 10^−4^, and p12 = 10^−5^, or approximately a 75% prior belief that a signal will only be observed in the GSCAN GWAS and < 0.01% prior belief in favour of colocalisation between the two traits at a given locus [35].

## Results

### Observational analyses

In total, 45 431 pre-menopausal (39 188 in UK Biobank, 2343 in EPIC and 3900 in EHBCCG) and 173 476 post-menopausal (160 363 in UK Biobank, 4371 in EPIC and 8742 in EHBCCG) women were included in this analysis. Table 1 presents characteristics of the study participants.

#### Oestrogens:

**Figure 1:**
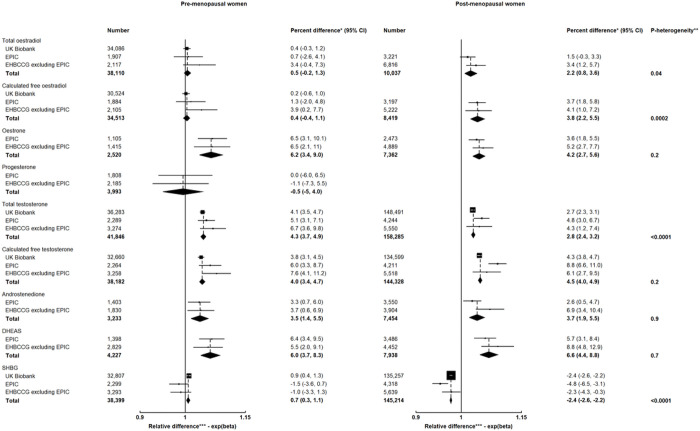
Associations of usual alcohol intake (per 10 g/day increment) with hormones and SHBG in pre- and post-menopausal women EPIC = European Prospective Investigation into Cancer and Nutrition; EHBCCG = Endogenous Hormones and Breast Cancer Collaborative Group DHEAS = Dehydroepiandrosterone sulphate; SHBG = Sex hormone binding globulin * Percent difference in concentrations of hormones and SHBG per 10 g/day increment in usual alcohol intake ** p-value for heterogeneity by menopausal status *** Relative difference in concentrations of hormones and SHBG per 10 g/day increment in usual alcohol intake

Alcohol intake was positively associated with concentrations of total and calculated free oestradiol in post-menopausal women but not in pre-menopausal women (p_heterogeneity by menopausal status_ = 0.04 for total oestradiol and 0.0002 for calculated free oestradiol). The concentrations were 2.2% (95% CI: 0.8%, 3.6%) and 3.8% (2.2%, 5.5%) higher, respectively, per 10 g/day increment in alcohol intake (approximately one drink/day) in post-menopausal women (Fig. 1).

Alcohol intake was positively associated with oestrone concentration in both pre- and post-menopausal women (Fig. 1). The concentrations were 6.2% (3.4%, 9.0%) and 4.2% (2.7%, 5.6%) higher, respectively, in pre- and post-menopausal women per 10 g/day increment in alcohol intake.

### Progesterone

Alcohol intake was not associated with progesterone concentration in pre-menopausal women (Fig. 1). No data were available for post-menopausal women.

### Androgens

Alcohol intake was positively associated with testosterone concentrations in both pre- and post-menopausal women (Fig. 1). Per 10 g/day increment in alcohol intake, the concentrations of total testosterone were 4.3% (3.7%, 4.9%) and 2.8% (2.4%, 3.2%) higher, respectively, in pre- and post-menopausal women, and those of calculated free testosterone were 4.0% (3.4%, 4.7%) and 4.5% (4.0%, 4.9%) higher, respectively. The associations for total testosterone were larger in pre-menopausal women (p_heterogeneity_<0.0001).

Similarly, alcohol intake was positively associated with concentrations of androstenedione and DHEAS in both pre- and post-menopausal women (Fig. 1). Per 10 g/day increment in alcohol intake, the concentrations of androstenedione were 3.5% (1.4%, 5.5%) and 3.7% (1.9%, 5.5%) higher, and those of DHEAS were 6.0% (3.7%, 8.3%) and 6.6% (4.4%, 8.8%) higher, respectively, in pre- and post-menopausal women. There were no differences in the associations by menopausal status.

### SHBG

Alcohol intake was positively associated with SHBG concentration in pre-menopausal women but inversely associated in post-menopausal women (p_heterogeneity_<0.0001; Fig. 1); the concentration was 0.7% (0.3%, 1.1%) higher in pre-menopausal women but was 2.4% (2.2%, 2.6%) lower in post-menopausal women per 10 g/day increment in alcohol intake.

### Associations by phase of the menstrual cycle in pre-menopausal women

**Figure 2:**
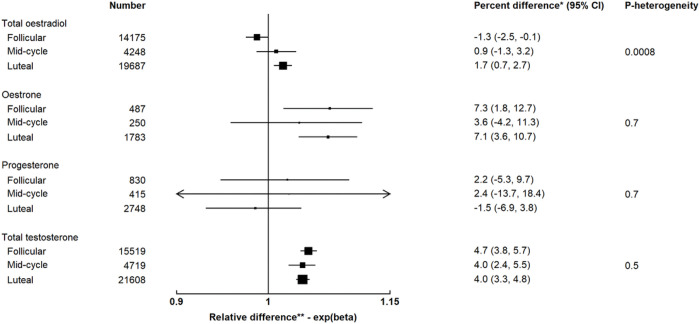
Associations of usual alcohol intake (per 10 g/day increment) with hormones and SHBG by phase of the menstrual cycle in pre-menopausal women * Percent difference in concentrations of hormones and SHBG per 10 g/day increment in usual alcohol intake ** Relative difference in concentrations of hormones and SHBG per 10 g/day increment in usual alcohol intake

Alcohol intake was inversely associated with total oestradiol (−1.3%; −2.5%, −0.1%) in the follicular phase but positively associated (1.7%; 0.7%, 2.7%) in the luteal phase (p_heterogeneity by cycle phase_=0.0008; Fig. 2). The associations for oestrone, progesterone and total testosterone did not differ by cycle phase.

### Associations by assay type

**Figure 3:**
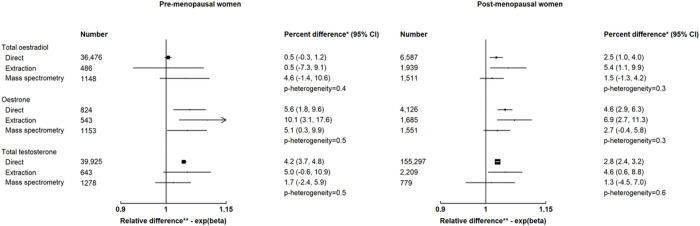
Associations of usual alcohol intake (per 10 g/day increment) with hormones and SHBG by assay type EPIC = European Prospective Investigation into Cancer and Nutrition; EHBCCG = Endogenous Hormones and Breast Cancer Collaborative Group DHEAS = Dehydroepiandrosterone sulphate; SHBG = Sex hormone binding globulin * Percent difference in concentrations of hormones and SHBG per 10 g/day increment in usual alcohol intake ** Relative difference in concentrations of hormones and SHBG per 10 g/day increment in usual alcohol intake

There were no differences in the associations by assay type for total oestradiol, oestrone and total testosterone (Fig. 3).

### Sensitivity analyses

The associations did not differ substantially when restricted to those who reported usual alcohol intake of < 15 g/day (Supplementary Figure S1), to those who reported intake of < 30 g/day (data not shown), or to samples collected during ovulatory cycles (data not shown).

## MR and colocalisation analyses

Effect estimates for the association of rs1229984 with alcohol intake and with concentrations of testosterone and SHBG are presented in Supplementary Table S6.

In MR analyses, a 10 g/day increment in genetically predicted alcohol intake was associated with higher concentrations of total testosterone (4.1%; 0.6%, 7.6%) and free testosterone (7.8%; 4.1%, 11.5%), and lower concentration of SHBG (−8.1%; −11.3%, −4.9%) (Table 2). Colocalisation analyses showed strong evidence in favour of a shared causal locus between alcohol intake and free testosterone (PP4 = 0.81) and SHBG (PP4 = 0.97) at *ADH1B* (Table 3, Supplementary Figure S2). MR results by menopausal status were not available.

## Discussion

In this meta-analysis involving over 45 000 pre-menopausal and 173 000 post-menopausal women, we found positive associations of alcohol intake with concentrations of sex hormones. We also found an inverse association with SHBG in post-menopausal women and some evidence of a small positive association in pre-menopausal women. The genetic analyses supported potential causal associations of alcohol intake with higher free testosterone and lower SHBG.

### Oestrogens

Alcohol may influence oestrogen concentrations by altering its metabolism and clearance [3], or by affecting aromatisation of androgens to oestrogens.[36] Earlier intervention studies reported an increase in concentrations of oestradiol and/or oestrone after alcohol intake in both pre- [3, 12] and post-menopausal women [13, 14], although some found a positive association only in those on hormone therapy [5, 6], or no significant effect (possibly due to small sample sizes) [9–11, 15].

Our observational analyses showed positive associations of alcohol with oestrone in both pre- and post-menopausal women and with oestradiol in post-menopausal women. Although the overall association with oestradiol in pre-menopausal women was not significant, we found a weak inverse association in the follicular phase and a weak positive association in the luteal phase. In contrast, in an earlier cross-over trial, daily alcohol intake for three consecutive menstrual cycles significantly increased plasma concentrations of ovulatory oestradiol but not follicular or luteal oestradiol [12].

The less conclusive findings observed for oestradiol in pre-menopausal women may be related to the challenges in measuring this hormone reliably; measurement based on a single serum sample may not reflect its long-term average as the hormone level varies substantially across the menstrual cycle. We standardised oestradiol concentrations for phase of the menstrual cycle in the observational analyses, but this may not be sufficient to account for all the variation [37]. Moreover, the studies included in the meta-analysis variably used forward or backward dating to define cycle phase when blood was collected. The positive association of alcohol with oestradiol in post-menopausal women was also of small magnitude, probably because the oestradiol concentration is low in this group and could be below or close to the lower limit of detection of some of the assays used, which is likely to have reduced statistical power; however, we found no differences in the association by assay type.

### Progesterone

Alcohol might influence progesterone concentration by altering its metabolism in the liver [9, 10], but the results from previous intervention studies have been mixed [9, 10, 12]. We found no association in pre-menopausal women overall as well as across three cycle phases, although our ability to detect any association may have been limited due to measurement errors associated with variations in the hormone level throughout the menstrual cycle.

### Androgens

Alcohol may influence androgen concentrations by altering their secretion from the ovaries and/or adrenal glands, or their metabolism in the liver [38]. Previous intervention studies reported an acute elevation in concentrations of one or more androgens after alcohol intake in both pre- [4, 7, 8] and post-menopausal women [14, 15], although others found no significant effect in pre-menopausal women possibly due to small sample sizes [9–12].

In this meta-analysis, we found positive associations of alcohol with testosterone, androstenedione and DHEAS in both pre- and post-menopausal women. The association with testosterone seemed to be of greater magnitude in pre-menopausal women even after restricting to those with intake of < 15 g/day, which might be due to biological differences or possibly due to differences in the accuracy of self-reported alcohol intake by menopausal status. The associations with androstenedione and DHEAS did not differ by menopausal status.

In the MR analyses, genetically predicted alcohol intake was positively associated with testosterone concentrations, with a larger effect on free testosterone compared to total testosterone. We observed strong colocalisation for alcohol intake at the *ADH1B* locus with free but not total testosterone. This raises the question as to whether or not alcohol has a direct causal effect on testosterone concentration, because the strong association with free testosterone could be related to the inverse association of alcohol intake with SHBG concentrations as discussed below.

### SHBG

Alcohol may influence SHBG concentrations by affecting hormonal balance [39], cytokine levels [40], hepatic synthesis/release or blood clearance [41, 42]. An earlier intervention study in pre-menopausal women showed a slight increase in SHBG concentration particularly in the mid-luteal phase [12] whereas another study of post-menopausal women found a decrease in concentration after 8–12 weeks of daily alcohol intake [13]; however, the results in both studies were not significant possibly due to small sample sizes.

Similarly in this meta-analysis, we found an inverse association of alcohol intake with SHBG in post-menopausal women and some evidence of a small positive association in pre-menopausal women; the latter was driven by the results from UK Biobank with no association in the other datasets, therefore this observation should be interpreted cautiously. The MR and colocalisation analyses at the *ADH1B* locus identified an inverse association, which is consistent with our observational findings because the GWAS for SHBG was conducted in the UK Biobank where the majority of women were post-menopausal. As SHBG binds testosterone to a greater degree than oestradiol, any reduction in SHBG caused by alcohol would be expected to have a bigger effect in increasing the bioavailable fraction of testosterone than oestradiol as observed in our analyses.

### Hormones and alcohol-induced breast carcinogenesis

Alcohol has been associated with an increased risk of several cancers, including female breast cancer [43, 44]. In the Million Women Study, with over 68 000 cases, there was a 12% increase in risk per 10 g/day increment in alcohol intake [44]. Our findings confirming the positive associations of alcohol intake with sex hormones, particularly their bioavailable fractions, support a probable role of sex hormones in alcohol-induced breast carcinogenesis. Given the published evidence supporting the effects of alcohol on both ER (oestrogen receptor) positive and negative breast cancer [45], it is possible that hormones interplay with acetaldehyde and other suggested mechanisms [46–48] influencing cancer risk.

### Strengths and limitations

To our knowledge, this is the largest study on this topic. Our meta-analysis involved over 45 000 pre-menopausal and 173 000 post-menopausal women, enabling us to undertake important subgroup analyses by menopausal status, cycle phase in pre-menopausal women and assay type. We additionally conducted MR and colocalisation analyses to support the observational results where possible.

Our main exposure, alcohol intake, was self-reported. While self-reported measures of alcohol intake may have reasonable levels of reliability and validity [49], underreporting is common particularly among those with very high intake [50], which could lead to overestimation of the magnitude of associations of reported alcohol intake with circulating hormones. The potential limitations related to oestradiol measurement have been discussed above; we have therefore not undertaken genetic analyses for this hormone. We used the female-specific genetic instruments for testosterone and SHBG, but were not able to undertake analyses separately for pre- and post-menopausal women. The genetic instruments for other important hormones included in the meta-analysis, such as progesterone, DHEAS and androstenedione were not publicly available. Finally, the study samples comprised mainly women of white European ancestry (e.g., approximately 95% in UK Biobank), limiting the generalisability of the results to other populations.

## Conclusions

Our meta-analysis confirmed positive associations of alcohol intake with sex hormones, including the more bioavailable fractions. There was also an inverse association with SHBG in post-menopausal women and some evidence of a small positive association in pre-menopausal women. Genetic analyses supported potential causal overall associations with higher free testosterone and lower SHBG. These associations are likely to contribute to the effect of alcohol on breast cancer risk.

## Figures and Tables

**Table 1:** Participant characteristics

	Pre-menopausal women			Post-menopausal women		
	UK Biobank (*n* = 39188)	EPIC (*n* = 2343)	EHBCCG excluding EPIC (*n* = 3900)	UK Biobank (*n* = 160363)	EPIC (*n* = 4371)	EHBCCG excluding EPIC (*n* = 8742)
Age at recruitment (years), mean (SD)	44.7 (2.7)	42.8 (4.3)	43.3 (4.5)	60.5 (5.3)	60.0 (5.4)	62.5 (7.0)
Cases, %		40.5	30.6		42.6	35.2
Usual alcohol intake (grams/day), median (IQR)	6.9 (13.3)	2.9 (11.5)	2.0 (8.0)	5.7 (12.9)	2.8 (10.6)	1.0 (7.0)
Usual alcohol intake (grams/day), mean (SD)	10.8 (12.9)	7.9 (11.7)	6.5 (11.7)	9.4 (11.4)	7.6 (11.2)	5.6 (10.6)
Current smoker, %	11.1	23.6	14.4	8.0	17.6	9.8
Body mass index (kg/m^2^), mean (SD)	26.3 (5.3)	24.7 (4.2)	24.7 (4.9)	27.3 (5.1)	26.5 (4.6)	26.7 (5.0)
Nulliparous, %	26.8	16.7	22.5	15.4	13.9	12.6
Past use of hormones, %	87.7	66.7	28.6	86.5	45.5	32.4
Age at menopause (years), mean (SD)				49.5 (5.7)	49.4 (4.6)	48.8 (5.2)
Natural menopause, %				84.0	96.7	68.1
Total oestradiol (pmol/L), median (IQR)	346.1 (365.4)	269.6 (230.3)	414.8 (289.4)		73.47 (63.31)	33.04 (41.66)
Calculated free oestradiol (pmol/L), median (IQR)	4.05 (4.20)	3.66 (3.18)	5.10 (3.46)		1.10 (1.12)	0.54 (0.64)
Oestrone (pmol/L), median (IQR)		312.7 (236.2)	307.0 (166.4)		138.34 (79.44)	88.76 (72.65)
Progesterone (nmol/L), median (IQR)^a^		3.72 (5.48)	42.77 (38.10)			
Total testosterone (nmol/L), median (IQR)	1.12 (0.71)	1.28 (0.99)	0.90 (0.52)	0.85 (0.74)	1.15 (0.86)	0.80 (0.62)
Calculated free testosterone (pmol/L), median (IQR)	12.53 (10.32)	16.38 (16.59)	10.70 (7.85)	10.50 (10.78)	17.18 (16.32)	11.13 (9.87)
Androstenedione (nmol/L), median (IQR)		4.41 (3.24)	4.02 (2.49)		2.88 (2.27)	2.19 (1.70)
DHEAS (nmol/L), median (IQR)		3314.9 (2365.9)	2794.0 (2206.7)		1917.8 (1705.9)	1951.0 (1990.0)
SHBG (nmol/L), median (IQR)	62.91 (38.02)	51.97 (36.68)	58.51 (38.00)	53.52 (33.59)	40.71 (31.90)	47.71 (31.70)

SD = Standard deviation

IQR = Interquartile range

DHEAS = Dehydroepiandrosterone sulphate

SHBG = Sex hormone binding globulin

a Luteal phase progesterone measured in EHBCCG excluding EPIC

**Table 2:** Mendelian randomization estimates, instrumented by rs1229984, for usual alcohol intake (per 10 g/day increment) with hormones and SHBG in women

	Percent difference (95%CI)	*P*
Total testosterone	4.1 (0.6, 7.6)	0.02
Free testosterone	7.8 (4.1, 11.5)	0.00003
SHBG	−8.1 (−11.3, −4.9)	0.000001

SHBG = Sex hormone binding globulin

**Table 3:** Posterior probabilities from colocalisation analyses for rs1229984

	PP0	PP1	PP2	PP3	PP4
Total testosterone	1.68E-58	0.910	1.08E-60	0.006	0.084
Free testosterone	3.46E-59	0.188	7.34E-61	0.004	0.808
SHBG	5.57E-60	0.030	2.56E-61	0.001	0.968

SHBG = Sex hormone binding globulin

PP0 = Posterior probability for hypothesis 0 (H0): no association with either trait (alcohol intake or testosterone/SHBG concentration)

PP1 = Posterior probability for H1: association with trait 1 (alcohol intake), not with trait 2 (testosterone/SHBG concentration)

PP2 = Posterior probability for H2: association with trait 2 (testosterone/SHBG concentration), not with trait 1 (alcohol intake)

PP3 = Posterior probability for H3: association with both traits (alcohol intake and testosterone/SHBG concentration), two distinct SNPs

PP4 = Posterior probability for H4: association with both traits (alcohol intake and testosterone/SHBG concentration), one shared SNP

## Data Availability

UK Biobank is an open access resource, and the study website https://www.ukbiobank.ac.uk/ has information on available data and access procedures. For EPIC, details of data access are at https://epic.iarc.fr/access/index.php. For EHBCCG, the principal investigators of each contributing study are responsible for access to the data. Full GWAS summary statistics from GSCAN are available at https://conservancy.umn.edu/handle/11299/201564. The instruments for concentrations of testosterone and SHBG were extracted from the OpenGWAS platform https://gwas.mrcieu.ac.uk/.

## Abbreviations

ADH1B: Alcohol dehydrogenase 1B
BMI: Body mass index
CPS-II: Cancer Prevention Study-II
DHEAS: Dehydroepiandrosterone sulphate
EHBCCG: Endogenous Hormones and Breast Cancer Collaborative Group
EPIC: European Prospective Investigation into Cancer and Nutrition
ER: Oestrogen receptor
GSCAN: GWAS and Sequencing Consortium of Alcohol and Nicotine Use
GWAS: Genome-wide association study
HRT: Hormone replacement therapy
LD: Linkage disequilibrium
MCCS: Melbourne Collaborative Cohort Study
MEC: Multiethnic Cohort
MR: Mendelian randomization
NHS-I: Nurses’ Health Study I
NHS-II: Nurses’ Health Study II
NYU WHS: New York University Women’s Health Study
OC: Oral contraceptives
ORDET: Study of Hormones and Diet in the Etiology of Breast Tumors
PLCO: Prostate, Lung, Colorectal, and Ovarian Cancer Screening Trial
SD: Standard deviation
SHBG: Sex hormone binding globulin
SOF: Study of Osteoporotic Fractures
UKCTOCS: United Kingdom Collaborative Trial of Ovarian Cancer Screening
WHI-OS: Women’s Health Initiative, Observational Study
